# Blood Brain Barrier Permeability Could Be a Biomarker to Predict Severity of Neuromyelitis Optica Spectrum Disorders: A Retrospective Analysis

**DOI:** 10.3389/fneur.2018.00648

**Published:** 2018-08-07

**Authors:** Ying Wang, Mingqin Zhu, Caiyun Liu, Jinming Han, Wenjuan Lang, Yang Gao, Chao Lu, Shuang Wang, Shuai Hou, Nannan Zheng, Dong Wang, Yang Chen, Yu Zhang, Hong-Liang Zhang, Jie Zhu

**Affiliations:** ^1^Department of Neurology and Neuroscience Center, The First Hospital of Jilin University, Changchun, China; ^2^Department of Life Sciences, The National Natural Science Foundation of China, Beijing, China; ^3^Department of Neurobiology, Care Sciences & Society, Karolinska Institutet, Stockholm, Sweden

**Keywords:** blood brain barrier, neuromyelitis optica spectrum disorders, aquaporin 4, albumin index, biomarker, Expanded Disability Status Scale

## Abstract

**Background:** Blood-brain barrier (BBB) pathology exists in neuromyelitis optica spectrum disorders (NMOSD). However, the clinical use of BBB permeability, such as predicting disease severity of NMOSD, has rarely been studied in a large cohort of patients.

**Objectives:** The current study explored the association between BBB permeability and clinical parameters in order to assess if BBB permeability could be a biomarker to predict disease severity and clinical characteristics of NMOSD.

**Methods:** Among 69 enrolled NMOSD patients, 47 with albumin index over 5 × 10^−3^ were assigned to the increased BBB permeability group, and the remaining 22 were to the normal BBB permeability group. Disease severity was assessed using the Expanded Disability Status Scale (EDSS).

**Results:** Patients in the increased BBB permeability group had significantly higher EDSS scores, anti-aquporin-4 immunoglobulin G titers, more dense cerebrospinal fluid protein concentrations, white blood cell counts, myelin basic protein levels and more dense complement 3 concentrations than found in the comparative normal BBB permeability group. The albumin index was positively correlated to the length of lesions in spinal cord.

**Conclusions:** BBB permeability was associated with clinical features, laboratory results and radiological data of NMOSD patients, and may be a potential biomarker to predict disease severity and clinical characteristics of NMOSD.

## Introduction

Neuromyelitis optica spectrum disorders (NMOSD) are immune-mediated disorders in the central nervous system (CNS), and anti-aquporin-4 (AQP4) immunoglobulin G (IgG) is one of the major autoimmune antibodies that contributes the pathogenesis of NMOSD (1). AQP4 is a water channel protein (2) mainly expressing on astrocytes, which are the components of blood brain barrier (BBB) (3). Disturbances of the BBB are implicated in NMOSD (4, 5). When anti-AQP4-IgG obtained from a NMO patient was administered to mice, lesions of perivascular astrocyte were observed, indicating the involvement of BBB (4). Shimizu et al. further found that anti-AQP4 IgG positive sera from NMO patients may disrupt BBB by reducing the expression of tight junction proteins (5). Notably, anti-AQP4-IgG may be not the only kind of autoantibody leading to the disruption of BBB (5, 6). Many molecules have been found to be associated with the disturbance of BBB, including glucose-regulated protein 78 autoantibody, vascular endothelial growth factor, CD40, IL-6, and so on (5, 7–9). However, the clinical role of BBB permeability, such as predicting clinical severity and characteristics, has rarely been studied in large cohorts of NMOSD patients. The current study retrospectively investigates whether BBB permeability evaluated by albumin index could be a biomarker to predict clinical severity and characteristics of NMOSD. The hypothesis is that BBB disturbance is associated with clinical features, disease severity, laboratory results, and radiological findings of NMOSD patients. Currently, anti-AQP4 IgG is a widely used diagnostic biomarker for NMOSD, which obtains high specificity (10, 11). Additionally, anti-AQP4-IgG is associated with the features of the spinal cord lesions in NMO (12). Nevertheless, the serum of one third of the NMOSD patients is anti-AQP4 IgG negative, which is a significant detriment of anti-AQP4 IgG to be a biomarker to monitor the disease course of NMOSD (13, 14). Although investigations have indicated that various kinds of molecules are involved in the development of NMOSD, including inflammatory molecules, genetic biomarkers, CNS proteins, etc., evidence on their clinical roles of monitoring disease course is still lacking (15).

Herein, we have systematically explored the association between BBB permeability and clinical parameters in a large cohort of NMOSD patients to exam whether BBB permeability could be a biomarker to predict clinical severity and characteristics of NMOSD. The objective of this effort is to deepen clinical understanding of BBB permeability in NMOSD.

## Methods

### Ethics statement

The study was approved by the Ethics Committee of the First Hospital of Jilin University. Although informed consent in writing was not available, no individually identifying personal information appeared.

### Study subjects

We enrolled 88 patients, each of whom had been admitted between 2013 and 2015 to Department of Neurology, the First Hospital of Jilin University, and also had fulfilled the international consensus diagnostic criteria for NMOSD (1). All of the patients were enrolled during an acute attack of NMOSD. Basic information, a description of clinical symptoms, results of laboratory examinations, as well as the magnetic resonance images of spinal cord were collected for all NMOSD patients. As 17 patients refused the lumbar puncture, BBB permeability data was available in 69 out of the 88 patients. Both cerebrospinal fluid (CSF) and blood samples of patients were obtained at the first day of hospitalization before treatment.

### Cell based assay for AQP4 analysis

Blood and CSF samples were collected from NMOSD patients at admission and titers for anti-AQP4-IgG were examined using a commercial cell-based assay kit (Euroimmun, Lübeck, Germany), as has been previously reported (16). Briefly, HEK 293 cells transfected with AQP4-M1 were seeded onto the slides, patient serum or CSF (30 μl) was applied to the slides, which were incubated for 30 min at room temperature. The slides were washed with 1 x PBS-Tween 20 for 5 min. After that, 25 μl fluorescein labeled anti-human globulin was added to each reaction field and incubated for 30 min at room temperature. The slides were washed for 5 min with 1 x PBS-Tween 20. The slides were then mounted onto cover glasses using embedding medium after they had been dried. The fluorescence was evaluated under the microscope. The titers of anti-AQP4 IgG in the samples were ranked from 0 (negative) to 3 (strong positive) by two clinicians according to the intensity of fluorescence.

### BBB permeability measurement

The BBB permeability was measured using albumin index, which is the albumin level ratio, CSF/serum, as previously reported (6, 17). The levels of albumin in the serum and CSF were measured using enzyme-linked immunosorbent assay kits (Debo, Shanghai, China). All procedures were performed according to the manufacturer's instructions. Briefly, the 96-well plate was pre-coated with albumin antibody. Pre-diluted blood or CSF samples and recombinant standard proteins were added to the plate (50 μL/well) for 30 min incubation at 37°C. Thereafter, the plates were washed four times with 300 μL of wash buffer, then were combined with developing solution. After a 20 min incubation at 37°C, 50 μL of stop solution was added to end the reaction. Optical density was determined at 490 nm by enzyme-labeled meter, and was further analyzed by Curve Export. The albumin concentration was calculated by extrapolation from the standard curve. A BBB disruption was defined as the albumin index more than 5.0 × 10^−3^.

### Disease severity assessment

The Expanded Disability Status Scale (EDSS), a widely used scoring system to evaluate the severity of NMOSD, was employed to assess the disability of enrolled patients (18). In this scale, visual, brainstem, pyramidal, cerebellar, sensory, bowl/bladder, cerebral functions, and ambulation were scored separately. The EDSS score was ranked by two clinicians the same day when CSF and blood were sampled.

### Statistical analysis

Statistical analyses were performed using the SPSS version 18.0 software (SPSS, IBM, West Grove, PA, USA). The normality was analyzed using Shapiro-Wilk test. For categorical data, Chi-square test was used for analyzing the difference between groups. Normally distributed data were analyzed using the Student *t* test. Non-normally distributed data were analyzed using Mann-Whitney U test. Correlation analysis was performed with the non-parametric Spearman's *rho* test. A two-sided *p* value < 0.05 was considered statistically significant.

## Results

### Occurrence of BBB disturbance among NMOSD patients

In the current study, BBB permeability was evaluated using the albumin index, and the data were collected from the records of inpatients. Sample description, i.e., clinical, laboratory and radiologic data, are showen in the Supplementary Table. Among the enrolled patients, BBB permeability data was available for 69 of the 88 patients. Increased BBB permeability (CSF albumin concentration/serum albumin concentration > 5 × 10^−3^) was observed in 68.12% (47/69) of the patients. Patients were assigned to two groups on the basis of BBB permeability, 47 of the 69 patients with albumin index over 5 × 10^−3^ to the increased BBB permeability group, and the remaining 22, whose albumin index was within the reference range, to the normal BBB permeability group.

### The increased BBB permeability group had a more severe disease course

**Figure 1:**
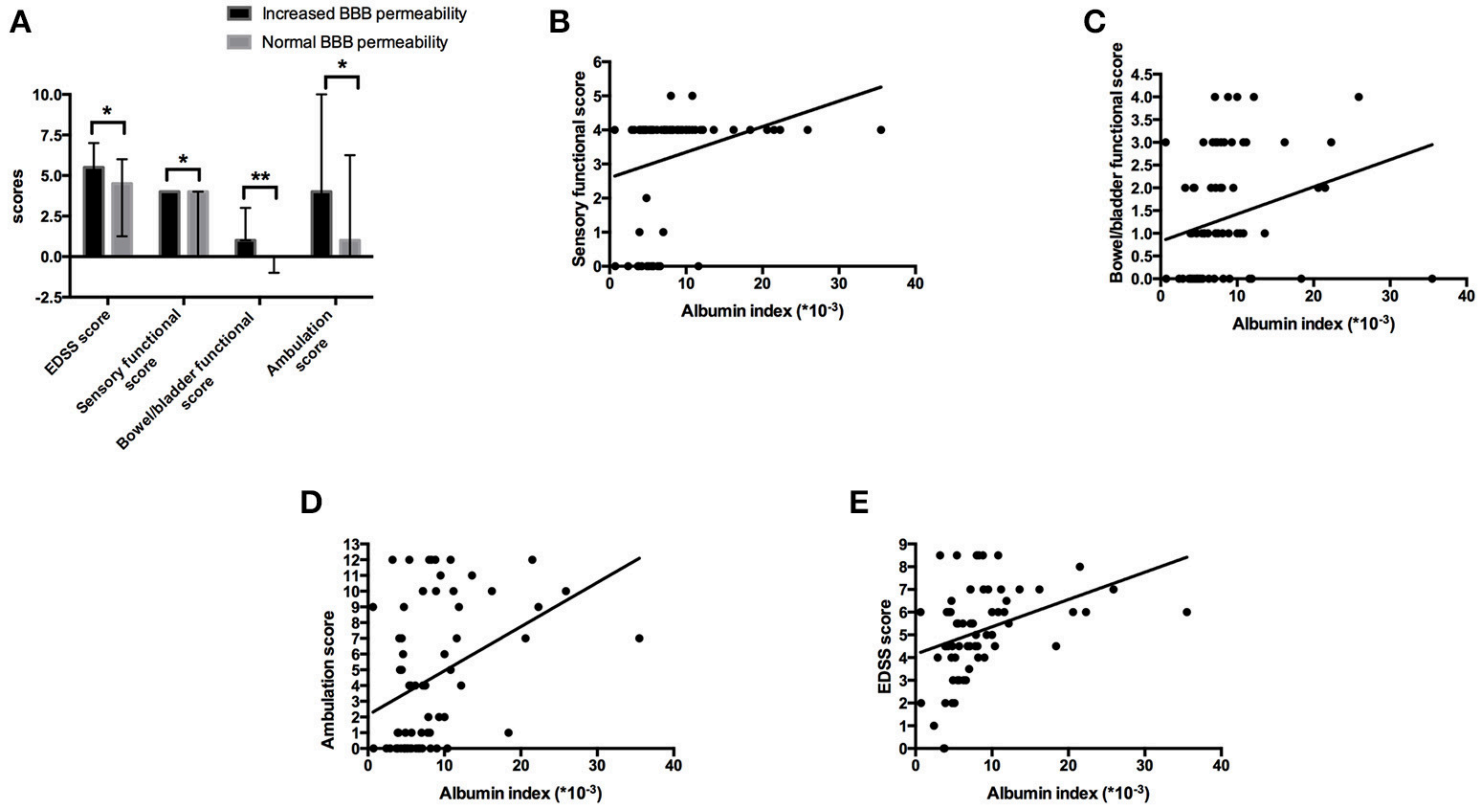
Increased BBB permeability was associated with more severe disease course. **(A)** Patients in the increased BBB permeability group suffered a more severe disease course. The median of EDSS score of the increased BBB permeability group was 5.5 with IQR of 4.5–7.0, while was 4.5 with IQR of 2.25–6 in the normal BBB permeability group (*p* = 0.028). Additionally, the increased BBB permeability group had significantly higher sensory functional score, bowel/bladder functional score and ambulation score (4 with IQR of 4-4, 1 with IQR of 1-3 and 4 with IQR of 1-10 vs. 4 with IQR of 0-4, 0 with IQR of 0-1, 1 with IQR of 0-6.25, *p* = 0.038, 0.001, 0.036). ^*^p < 0.05, ^**^p < 0.01. **(B–E)** The albumin index was positively correlated to sensory functional score, bowel/bladder functional score, ambulation score and EDSS score (*r* = 0.372, *p* = 0.002; *r* = 0.420, *p* = 0.000; *r* = 0.434, *p* = 0.000; *r* = 0.449, *p* = 0.000).

For the basic information of patients, the male/female ratio, median of age (years) and duration of hospitalization (days) in the increased BBB permeability group were 25.53%, 52 with IQR of 45-58, and 17 with IQR of 12-22, while the counterparts in the normal BBB permeability group were 18.18%, 44 with IQR of 28.5-54.5, and 16.5 with IQR of 12-22.25, respectively. The mean age of NMOSD patients in the increased BBB permeability group was significantly higher than that of the normal BBB permeability group (*p* = 0.038). The age of the patients was also positively correlated with the albumin index (*r* = 0.329, *p* = 0.006). With regard to the disease severity, the increased BBB permeability group scored significantly higher on EDSS than the normal BBB permeability group (*p* = 0.028) (Figure 1A). In addition, functional scores for sensation, bowel/bladder function and ambulation distinguished between the two groups (Figure 1A), and were positively correlated to albumin index (Figures 1B–E). The functional scores measuring visual, brainstem, pyramidal and cerebellar capacities, on the basis of EDSS, did not significantly differentiate between the two groups (0 with IQR of 0-3, 0 with IQR of 0-0, 3 with IQR of 1-3, and 0 with IQR of 0-0 vs. 0 with IQR of 0-2, 0 with IQR of 0-1, 1.5 with IQR of 0-3, and 0 with IQR of 0-0, *p* = 0.256, 0.229, 0.067, and 0.481).

### BBB permeability was associated with anti-AQP4-IgG titer and other laboratory results

**Figure 2:**
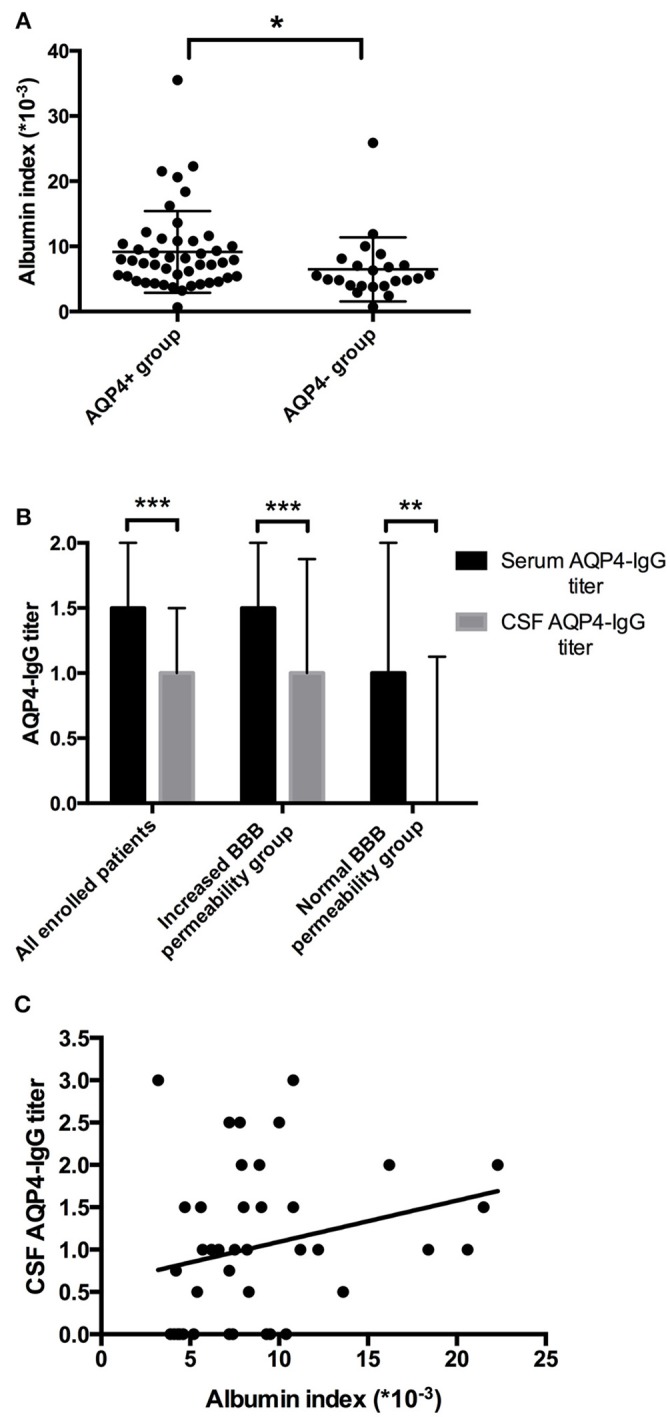
Elevated BBB permeability was associated with higher anti-AQP4-IgG levels. **(A)** The BBB permeability was increased in the AQP4+ group. The mean of albumin index in the AQP4+ group was 9.15 (× 10^−3^) with SD of 6.26 (× 10^−3^), while 6.50 (× 10^−3^) with SD of 4.92 (× 10^−3^) in the AQP4- group. **(B)** Anti-AQP4-IgG titer was higher in serum than in CSF. For all 69 enrolled patients, the median anti-AQP4 IgG titers were 1.5 with IQR of 1-2, and 1 with IQR of 0-1.5 in serum and CSF (*p* = 0.000). In the increased BBB permeability group, the median serum and CSF anti-AQP4-IgG titers were 1.5 with IQR of 1-2 and 1 with IQR of 0.5-1.875 (*p* = 0.000), while 1 with IQR of 0.75-2 and 0 with IQR of 0-1.125 in the normal BBB permeability group (*p* = 0.006). **(C)** The albumin index was positively correlated to the CSF anti-AQP4-IgG titer (*r* = 0.351, *p* = 0.025). ^*^p < 0.05, ^**^p < 0.01, ^***^p < 0.001.

Sixty-nine patients were further divided into anti-AQP4-IgG positive (AQP4+) group and anti-AQP4-IgG negative (AQP4-) group. Forty-six NMOSD patients with anti-AQP4-IgG positive in serum and/or CSF were included in AQP4+ group, the other 23 patients, whose anti-AQP4-IgG were negative in neither serum or CSF, fell into AQP4- group. Within AQP4+ group, serum anti-AQP4-IgG was positive in 45 patients while CSF anti-AQP4-IgG was positive among 31 patients. The female/male ratio, medians of age and days of hospitalization (IQR) were 37/9, 53 (45.75–58) and 17 (11.75–21.25) in AQP4+ group and were 16/7, 41 (29–53) and 17 (13–23) in AQP4– group. Interestingly, BBB disturbance was observed in both AQP4+ and AQP4– groups. However, the proportion of patients who had BBB disturbance was significantly higher in AQP4+ group than in AQP4– group (74.47% vs. 50.00%, *p* = 0.045). The albumin index was also significantly increased in the AQP4+ group compared to that in the AQP4– group (Figure 2A).

**Figure 3:**
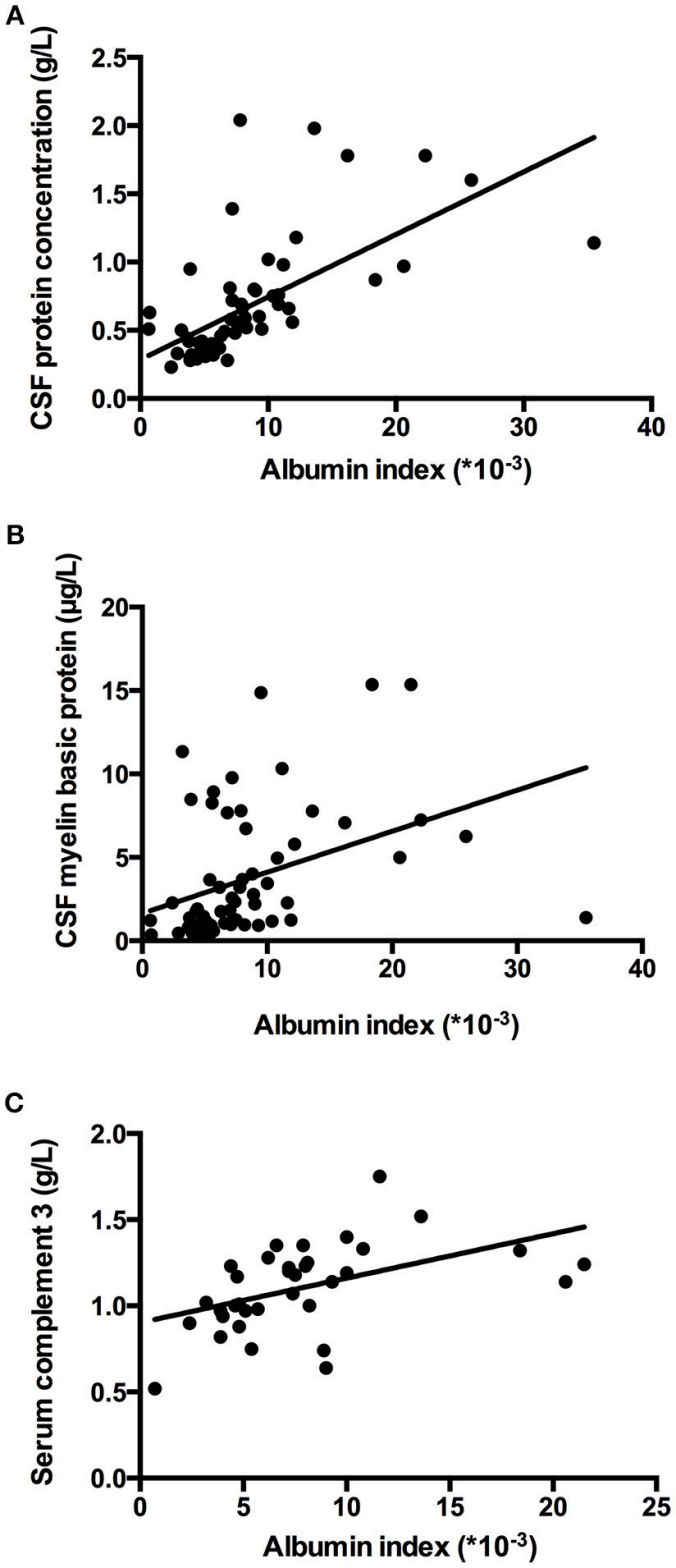
Albumin index was correlated to laboratory parameters. **(A–C)** Albumin index was positively correlated to the CSF protein concentration, CSF myelin basic protein level and serum C3 concentration (*r* = 0.633, *p* = 0.000; *r* = 0.386, *p* = 0.001; *r* = 0.484; *p* = 0.003).

The relation of the titer of anti-AQP4-IgG to BBB permeability was further investigated in the AQP4+ group. The titer of anti-AQP4-IgG is much higher in serum than in CSF (Figure 2B); and the CSF anti-AQP4-IgG titer was significantly higher in the increased BBB permeability group than in the normal BBB permeability group (*p* = 0.041). The albumin index was positively correlated to the CSF anti-AQP4-IgG titer (*r* = 0.351, *p* = 0.025) (Figure 2C). However, there was no significant difference of serum anti-AQP4-IgG titer between the two groups (*p* = 0.205). Except for anti-AQP4-IgG, other laboratory parameters also did not distinguish between the two groups (Table 1). For CSF examination results, the protein concentration, white blood cell count and myelin basic protein level were significantly higher in the increased BBB permeability group (Table 1). In blood examination, myelin basic protein level and complement component 3 (C3) concentration were elevated in the increased BBB permeability group (Table 1). In addition, the CSF protein concentration, CSF myelin basic protein level and serum C3 concentration were positively correlated with the albumin index (Figure 3). Other negative results with regard to laboratory examinations are also presented in Table 1.

**Table 1:** Laboratory results of two groups of patients.

**CSF examination**	**Increased BBB permeability**	**Normal BBB permeability**	***p* values**
CSF protein (g/L)^b^	0.78(0.47)	0.40(0.16)	0.000
CSF glucose (mmol/L)^b^	4.02(1.58)	3.60(0.67)	0.136
CSF white blood cell (× 10^6^/L)^b^	27.33(35.12)	14.76(11.34)	0.038
Polynuclear cells (%) ^b^	19.91(16.69)	19.25(20.77)	0.914
Mononuclear cells (%)^b^	75.75(21.23)	80.75(20.77)	0.488
IgG concentration (mg/L)^b^	84.82(89.72)	57.05(35.50)	0.306
Myelin basic protein (μg/L)^b^	4.56(4.06)	1.84(2.77)	0.003
Myelin basic protein antibody (OD value)^b^	0.17(0.33)	0.18(0.17)	0.861
**BLOOD EXAMINATION**			
Myelin basic protein (μg/L)^b^	6.40(5.20)	2.67(3.21)	0.001
Myelin basic protein antibody (OD value)^b^	0.68(0.90)	0.75(0.98)	0.800
White blood cell (× 10^9^/L)^b^	7.33(2.41)	10.04(9.39)	0.220
Thyroglobulin (IU/ml)^b^	215.82(810.88)	122.34(192.11)	0.710
Thyroid peroxidase antibody (IU/ml)^b^	123.37(336.78)	72.28(110.34)	0.636
Creative protein (mg/L)^b^	7.13(17.79)	1.98(2.55)	0.307
Complement 3 (g/L)^b^	1.18(0.25)	0.95(0.19)	0.011
Complement 4 (g/L)^b^	0.22(0.07)	0.18(0.06)	0.069

b *Mean (SD)*.

### BBB permeability was associated with radiographic presentation of NMOSD patients

We found that the number of segments of spinal cord that were involved was significantly higher in the increased BBB permeability group than in the normal BBB permeability group (*p* = 0.007). The median of the number of segments of spinal cord involved was 7 with IQR of 4-12 in the increased BBB permeability group; and a median of 3 with IQR of 2-8 in the normal BBB permeability group. Albumin index was positively correlated to number of segments of spinal cord involved (*p* = 0.001, *rs* = 0.391). No significant difference of lesion distribution showed up between two groups. The proportions of patients with cervical cord and thoracic cord involvement were 63.83 and 78.72% in the increased BBB permeability group, the counterparts in the normal BBB permeability group were 59.09 and 59.09% (*p* = 0.705, 0.089).

## Discussion

We have investigated possible associations between BBB permeability and clinical parameters in a large cohort of NMOSD patients and found that BBB disturbance appeared in a high proportion of NMOSD patients. These patients had been afflicted by more severe disease course. Additionally, they had higher anti-AQP4-IgG titer, CSF myelin basic protein concentration, and serum C3. The patients in the increased BBB permeability group also had longer spinal cord lesion than the patients in the normal BBB permeability group. Thus, BBB permeability could be a useful biomarker to monitor disease severity for NMOSD patients.

NMOSD is a demyelinating disease in CNS with high morbidity and mortality (19, 20). With the results of this investigation new considerations on the biomarkers of NMOSD are raised. Firstly, we find that the albumin index correlates with the severity of disease, as well as with the length of spinal cord lesion. Previous studies have identified other biomarkers of NMOSD, with various limitations (15). Anti-AQP4 IgG is considered to be a major molecule contributing to the pathogenesis of NMOSD, and its use in diagnosis of NMOSD could bring high specificity (10, 11). However, anti-AQP4 IgG is absent in the serum of one-third of NMOSD patients (13, 14), which limits the use of anti-AQP4 IgG to monitor the disease course of NMOSD. In anti-AQP4 IgG negative patients, myelin oligodendrocyte glycoprotein IgG is considered as an important biomarker (21). Other molecules of various kinds are also found to play important roles in the development of NMOSD (15). However, the association between these molecules and the clinical features of NMOSD has been rarely studied. Our study addresses albumin index as a predictor of disease severity and clinical characteristics of NMOSD, and we cite several advantages. Unlike anti-AQP4 IgG, the value of albumin index is available in every NMOSD patient. As the BBB is the target of anti-AQP4 IgG and other types of antibodies (5, 7–9), albumin index is more specific and relevant to the disease than various other kinds of molecules, such as cytokines and CNS proteins (15). Tomizawa et al. reported similar results and identified an association between albumin index and clinical severity of NMO (17). However, the patients in their study were NMO patients, not NMOSD patients; and only 21 patients were recruited. Furthermore, in their effort to evaluate the severity of disease, only EDSS scores was utilized in the study (17). Whereas, in our study more clinical parameters were included. Jarius et al. found that albumin index was positively correlated to the length of spinal cord lesion, which supports our results (22). However, their research was merely conducted on anti-AQP4 IgG positive NMO patients. In the contrast, our study includes both anti-AQP4 IgG positive and anti-AQP4 IgG negative NMOSD patients. Secondly, we find that BBB permeability is positively correlated to serum anti-AQP4-IgG titer. Whether merely serum anti-AQP4 IgG is sufficient to induce BBB disturbance is controversial (4, 6). Asgari et al. reported the BBB disturbance in mice after administering with anti-AQP4-IgG from NMO patients (4). However, Akaza et al. found that anti-AQP4-IgG was not associated with BBB damage (6). Our results further support the first perspective, and establish the quantitative link between BBB permeability and serum anti-AQP4-IgG titer. The negative results from Akaza et al. may be due, again, to a small sample (39 patients), different recruitment criteria from ours, and distinct method to assess BBB permeability (6). As the target of the anti-AQP4-IgG is AQP4 on the membrane of astrocytes, which compose of BBB (3), the correlation between anti-AQP4-IgG titer and BBB permeability could be explained. Although the underlying mechanism for anti-AQP4-IgG-mediated BBB disturbance is unclear, it has been demonstrated that the binding of anti-AQP4-IgG with AQP4 may increase the BBB permeability via multiple neuropathogenic mechanisms including complements activation and antibody dependent cell mediated cytotoxicity (4, 5). Interestingly, we find that BBB disruption also appeared in anti-AQP4-IgG negative NMOSD patients. This indicates that multiple mechanisms of BBB disturbance may exist. Except for anti-AQP4-IgG, cumulative evidence has disclosed that various immune molecules take part in the pathogenesis of NMOSD, such as myelin oligodendrocyte glycoprotein, AQP1-IgG, and so on (15, 23). Additionally, except for serum anti-AQP4-IgG, our study also showed that CSF anti-AQP4-IgG titer was positively correlated to the albumin index, which could result from the disturbance of BBB. Finally, we found that myelin basic protein and C3 levels are associated with BBB permeability. Myelin basic protein is a structural myelin protein, the concentration of which could indicate the severity of neuron impairment (24). It provides further evidence to support that BBB permeability is associated with the severity of NMOSD. Increased myelin basic protein level in CSF could also result from BBB disturbance. The correlation between BBB permeability and C3 levels implies that C3 modulates the pathogenesis of BBB damage.

This study deepens our understanding of NMOSD. We found that BBB permeability could be a biomarker to predict disease severity and clinical characteristics of NMOSD. Clinicians might consider this as a biomarker in their effort to provide intensive care to the NMOSD patients with high BBB permeability to improve the outcome of the patients. The disturbance of BBB is one of the earliest pathological changes in multiple sclerosis (25), which is another immune-mediated demyelination disease in CNS. Our investigation suggests that BBB damage might also be the starting point of NMOSD, which shares several similarities with multiple sclerosis.

Our study has limitations. Firstly, the follow-up data of the enrolled patients are lacking. The association between BBB permeability and prognosis was not analyzed. Secondly, BBB permeability was measured only once during the disease course. The evidence would be more convincing if the BBB permeability was detected at different stages of the disease for each patient. Thirdly, BBB permeability could be influenced by various factors, such as drugs, age, infection, etc. Fourthly, myelin oligodendrocyte glycoprotein IgG is an important biomarker among anti-AQP4 IgG negative NMOSD patients. However, as only four patients were myelin oligodendrocyte glycoprotein IgG positive, the association between myelin oligodendrocyte glycoprotein IgG and BBB permeability has not been able to be analyzed. For future investigations, the associations between BBB permeability and outcome of NMOSD as well as myelin oligodendrocyte glycoprotein IgG should be explored. A prospective study could investigate variation in BBB permeability at different disease phases. Studies focusing on the mechanisms of BBB damage are warranted as well.

## Data availability statement

Datasets are available on request.

## Author contributions

YW and MZ design of work, acquisition, analysis and interpretation of data, drafting the work. CyL, JH, WL, YG, CL, SW, SH, NZ, DW, YC, and YZ acquisition, analysis and interpretation of data. H-LZ revising the manuscript. JZ design of work, analysis and interpretation of data, revising the manuscript, and provide approval for publication.

### Conflict of interest statement

The authors declare that the research was conducted in the absence of any commercial or financial relationships that could be construed as a potential conflict of interest.
