# Supplementary material for: Blood Brain Barrier Permeability Could Be a Biomarker to Predict Severity of Neuromyelitis Optica Spectrum Disorders: A Retrospective Analysis
Source: Front Neurol. 2018 Aug 7;9:648. doi: 10.3389/fneur.2018.00648 (PMC6090143; doi:10.3389/fneur.2018.00648)
Supplement: Supplementary file 1 [file Table_1.docx]

**Table 1. Description of enrolled NMOSD patients**

| Basic information | Values | Reference values |
| --- | --- | --- |
| Gender (female/male) | 23.86% | N/A |
| Age (years)^a^ | 51 (36.25-57) | N/A |
| Duration in hospital (days)^a^ | 16.5 (13-20.75) | N/A |
| EDSS score ^a^ | 4.5 (4-6) | 0 |
| Visual function ^a^ | 1 (0-3) | 0 |
| Brainstem function ^a^ | 0 (0-0) | 0 |
| Pyramidal function ^a^ | 3 (1-3) | 0 |
| Cerebellar function ^a^ | 0 (0-0) | 0 |
| Sensory function ^a^ | 4 (1-4) | 0 |
| Bowel/bladder function ^a^ | 1 (0-2) | 0 |
| Cerebral function ^a^ | N/A | 0 |
| Ambulation ^a^ | 1 (0-8.5) | 0 |
| Physical signs |  |  |
| Optic nerve involvement | 38.64% | Absence |
| Other cranial nerve involvement | 18.18% | Absence |
| Hypermyotonia | 16.91% | Absence |
| Paralysis | 71.59% | Absence |
| Hyperreflexia | 68.18% | Absence |
| Pathologic reflexes | 61.93% | Absence |
| Sensory deficits | 71.59% | Absence |
| Blood examination results |  |  |
| AQP4 positive rate | 64.77% | 0 |
| AQP4 titer (among AQP4 positive patients) ^a^ | 1.5 (1-2) | N/A |
| Myelin oligodendrocyte glycoprotein antibody | 7.02% | N/A |
| Myelin basic protein (μg/L) ^b^ | 4.86 (4.86) | <2.5 |
| Myelin basic protein antibody (OD value)^b^ | 0.72 (0.93) | <0.75 |
| Erythrocyte sedimentation rate (mm/h) ^b^ | 19.56 (19.95) | 0-15 |
| White blood cell (X 10^9^/L)^b^ | 8.15 (5.46) | 4-10 |
| Thyroglobulin (IU/ml)^b^ | 205.00 (617.65) | <115.0 |
| Thyroid peroxidase antibody (IU/ml)^b^ | 108.05 (268.60) | <35.0 |
| C-reative protein (mg/L)^b^ | 5.64 (14.37) | 0-3.5 |
| Complement 3 (g/L)^b^ | 1.13 (0.25) | 0.9-1.8 |
| Complement 4 (g/L)^b^ | 0.22 (0.07) | 0.1-0.4 |
| CSF examination results |  |  |
| AQP4 titer ^a^ | 1 (0-1.5) | 0 |
| CSF protein (g/L) ^b^ | 0.78 (0.81) | 0.2-0.45 |
| CSF glucose (mmol/L) ^b^ | 3.94 (1.41) | 2.5-4.5 |
| CSF white blood cell (X 10^6^/L)^b^ | 23.01 (29.21) | 0-10 |
| Polynuclear cells (%) ^b^ | 18.85% (17.20) | N/A |
| Mononuclear cells (%)^b^ | 76.19% (23.08) | N/A |
| BBB permeability (X 10^-3^)^b^ | 8.25 (5.95) | <5.0 |
| IgG concentration (mg/L)^b^ | 85.21 (98.08) | 0-34.0 |
| Myelin basic protein (μg/L)^b^ | 3.52 (3.78) | <3.5 |
| Myelin basic protein antibody (OD value)^b^ | 0.20 (0.31) | <0.65 |
| Magnetic resonance images of spinal cord |  |  |
| Number of segments of spinal cord involved ^a^ | 3 (3-10) | 0 |
| Cervical cord involved ^a^ | 61.36% | Absence |
| Thoracic cord involved ^a^ | 69.32% | Absence |
| Lumbar cord involved ^a^ | 1.15% | Absence |
| Co-existing of other autoimmune disease | 14.8% | Absence |

^a^Median (IQR)

^b^Mean (SD)
